# Exploring the FGFR3-related oncogenic mechanism in bladder cancer using bioinformatics strategy

**DOI:** 10.1186/s12957-017-1125-4

**Published:** 2017-03-20

**Authors:** Wei Cao, Enguang Ma, Li Zhou, Tan Yuan, Chunying Zhang

**Affiliations:** 1 Department of Urinary Surgery, The Second Affiliated Hospital of Harbin Medical University, 150086 Harbin, Heilongjiang province China; 2 Department of Urinary Surgery, Harbin First Hospital, 150010 Harbin, Heilongjiang province China

**Keywords:** Bladder cancer, Fibroblast growth factor receptor 3, Differentially expressed gene, Protein–protein interaction network, Prognostic maker

## Abstract

**Background:**

Aberrant activation of fibroblast growth factor receptor 3 (FGFR3) is frequently observed in bladder cancer, but how it involved in carcinogenesis is not well understood. The current study was aimed to investigate the underlying mechanism on the progression of bladder cancer.

**Methods:**

The GSE41035 dataset downloaded from Gene Expression Omnibus was used to identify the differentially expressed genes (DEGs) between bladder cancer cell line RT112 with or without depletion of FGFR3, and gene ontology enrichment analysis was performed. Then, FGFR3-centered protein–protein interaction (PPI) and regulatory networks were constructed. Combined with the data retrieved from GSE31684, prognostic makers for bladder cancer were predicted.

**Results:**

We identified a total of 2855 DEGs, and most of them were associated with blood vessel morphogenesis and cell division. In addition, KIAA1377, POLA2, FGFR3, and EPHA4 were the hub genes with high degree in the FGFR3-centered PPI network. Besides, 17 microRNAs (miRNAs) and 6 transcriptional factors (TFs) were predicted to be the regulators of the nodes in PPI network. Moreover, CSTF2, POLA1, HMOX2, and EFNB2 may be associated with the prognosis of bladder cancer patient.

**Conclusions:**

The current study may provide some insights into the molecular mechanism of FGFR3 as a mediator in bladder cancer.

## Background

Bladder cancer, which refers to the cancer arising from the epithelial lining of urinary bladder, is one of the most frequent malignant cancers in the world. Its incidence has steadily increased and ranked as the sixth most common form of cancer in 2013 [1]. Though not fully known, the occurrence and development of bladder cancer is closely associated with multiple environmental factors, such as drinking, smoking, and contacting with chemical products, as well as many genetic factors [2].

Fibroblast growth factor receptor 3 (FGFR3), which belongs to the family of tyrosine kinase (RTK), is responsible for the FGF signal transduction [3]. FGFR3 signaling plays regulatory roles in cell proliferation, differentiation, and survival [4]. It is implicated in diverse physiologic and pathologic processes. Previous study revealed that FGFR3 is linked to the development of bladder cancer and FGFR3 overexpression and mutations are frequent events in patients with bladder cancer [5]. Moreover, it has been proved that bladder cancer cell proliferation in culture is inhibited when the activation of FGFR3 was blocked [6]. There is no doubt that FGFR3 takes vital roles in the process of tumorigenesis, but how FGFR3 signaling contributes to carcinogenesis is still unclear.

In current study, we reanalyzed the dataset GSE41035 (https://www.ncbi.nlm.nih.gov/geo/query/acc.cgi?acc=GSE41035), which provided the gene expression profiles of bladder cancer cell line RT112 with or without depletion of FGFR3, to identify the differentially expressed genes (DEGs) upon loss of FGFR3 in bladder cancer. Based on this dataset, Du et al. (another contributor of GSE41035) have documented that FGFR3 can stimulate stearoyl CoA desaturase 1 activity to promote bladder tumor growth [7]. Protein–protein interaction (PPI) and regulatory networks related to FGFR3 were constructed to investigate the possible mechanisms underlying the oncogenic role of FGFR3 in bladder cancer. Besides, we predicted the potential prognostic makers from the DEGs in the FGFR3-centered PPI network for bladder cancer combined with the data retrieved from the dataset GSE31684 [8].

## Methods

### Affymetrix microarray data

In current study, gene expression data of GSE41035 (https://www.ncbi.nlm.nih.gov/geo/query/acc.cgi?acc=GSE41035) and GSE31684 [8] were downloaded from Gene Expression Omnibus (GEO) database (http://www.ncbi.nlm.nih.gov/geo/), respectively. Both of these two datasets were sequenced on [HG-U133_Plus_2] Affymetrix Human Genome U133 Plus 2.0 Array. In GSE41035 uploaded by Modrusan et al., total of 24 cell samples, ranging from GSM1007032 to GSM1007055, were included. In this dataset, RT112 cells, a bladder cancer cell line, were transduced with a doxycycline-inducible control EGFP short hairpin (shRNA) or three independent FGFR3 shRNAs and then treated with or without doxycycline for depletion of FGFR3 protein. Consequently, gene expression alterations induced by FGFR3 loss would be identified by microarray analysis. The dataset GSE31684 uploaded by Riester et al. contained the gene expression patterns associated with clinical and prognostic variables of 93 bladder cancer patients managed by radical cystectomy. For these 93 patients, the median ages were ranged from 32.1 to 91.1 years and the male/female ratio was 68/25. Meanwhile, 95% of them were transitional cell carcinoma (TCC), and the remains were TCC/squamous. In addition, the radical cystectomy stages of them were 5 cases of pTa, 10 cases of pT1, 17 cases of pT2, 42 cases of pT3, and 19 cases of pT4, and the follow-up times of them were ranged from 1 to 175 months. This study was approved by Memorial Sloan-Kettering Cancer Center Institutional Review Board.

### Data preprocessing and DEG identification

RankProd is a simple and rapid method for DEG identification, which is based on the rank of gene expression and reliably utilized to explore difference of dataset with small sample size. After normalized by robust multi-array average (RMA) [9], the probe-level data in CEL files were converted into expression measures. Then, RankProd [10] was applied to identify the differentially expressed genes between the FGFR3-depleted RT112 cells and the control cells. False discovery ratio (FDR) <0.05 was set as the threshold.

### GO functional enrichment analysis

Gene Ontology (GO) is a tool for functional annotation of large-scale genomic data [11]. In the current study, we used GO function [12] to identify the over-represented GO biology processes among the DEGs. *p* value <0.05 was chosen as cutoff criterion.

### Construction of FGFR3-centered PPI network

The PPI data was collected from the Human Protein Reference Database (HPRD) [13], a centralized resource for information about human proteins, their interactions with other human proteins, and protein–disease relationships. The PPI network was generated by mapping the previously identified DEGs to the PPI data. FGFR3 was employed as a query node to construct the FGFR3-centered PPI network. First, the direct interacting DEGs with FGFR3 were selected, and then, the neighbors of these DEGs were also selected. The subnetwork was generated based on these proteins and visualized by Cytoscape [14].

### Construction of regulatory network

The experimentally validated dataset of human miRNAs/targets and transcriptional factors (TFs)/targets were respectively retrieved from miRTarBase [15], a database listing the miRNA–mRNA interactions collected from the literatures in PubMed, and TRANSFAC (TRANScription FACtor database) [16], a manually curated database of eukaryotic transcription factors, their genomic binding sites, and DNA binding profiles. Based on these two databases, miRNAs, which have regulatory targets among DEGs, and TFs included in DEGs were screened. The regulatory network associated with the genes in the above sub-PPI network was constructed using these data and visualized by Cytoscape [14].

### Exploring the potential prognostic makers for bladder cancer

The gene expression profiles and prognostic variables were retrieved from GSE31684 [8]. According to the median expression values of the genes in FGFR3-centered PPI network, patients with bladder cancer were divided into high- and low-expression groups. Prognosis-related genes were predicted by Cox regression model based on univariate analysis and multivariate analyses. Survival analysis was conducted by using the bioconductor splines package and survival package in R. Log-rank test was applied for comparison, and *p* value <0.05 was chosen as the threshold.

## Results

### Identification of DEGs

Genes that were differentially expressed after doxycycline induction (FDR <0.05) in the control cell line were considered as the DEG1 group, and those in 3 FGFR3-depleted cell lines were considered as the DEG2 group. Finally, we identified a total of 1428 upregulated and 1427 downregulated genes in the DEG2 group while there were only 28 upregulated and 58 downregulated genes in the DEG1 group.

### GO enrichment analysis

To determine the potential processes mediated by the DEG2 group, we separately mapped the upregulated and downregulated to the GO BP database. With *p* value <0.05 as the threshold, 5 GO terms were over-represented by the upregulated genes (Table 1 (a)) and the most significant one was regulation of transcription, DNA dependent (GO:0006355) with *p* value = 1.50E−06. The others were negative regulation of biological process (GO:0048519, *p* value = 1.69E−06), response to type I interferon (GO:0034340, *p* value = 1.42E−05), blood vessel morphogenesis (GO:0048514, *p* value = 2.42E−05), and epithelium development (GO:0060429, *p* value = 0.000125). However, 85 GO terms were enriched among the downregulated genes. Table 1 (b) lists the top 5 terms, from which we found that these terms mainly associated with cell division and carboxylic acid metabolic process.

**Table 1:** The enriched biological processes by the differentially expressed genes

GO term	Biological process_name	Count	*p* value
a) Upregulated genes			
“GO:0006355”	“regulation of transcription, DNA dependent”	260	1.50E−06
“GO:0048519”	“negative regulation of biological process”	282	1.69E−06
“GO:0034340”	“response to type I interferon”	17	1.42E−05
“GO:0048514”	“blood vessel morphogenesis”	56	2.42E−05
“GO:0060429”	“epithelium development”	67	0.000125
b) Downregulated genes			
“GO:0007067”	“mitosis”	132	0
“GO:0007346”	“regulation of mitotic cell cycle”	64	0
“GO:0051301”	“cell division”	107	0
“GO:0019752”	“carboxylic acid metabolic process”	145	4.31E−13
“GO:0051320”	“S phase”	68	2.78E−12

### FGFR3-cented PPI network

**Fig. 1:**
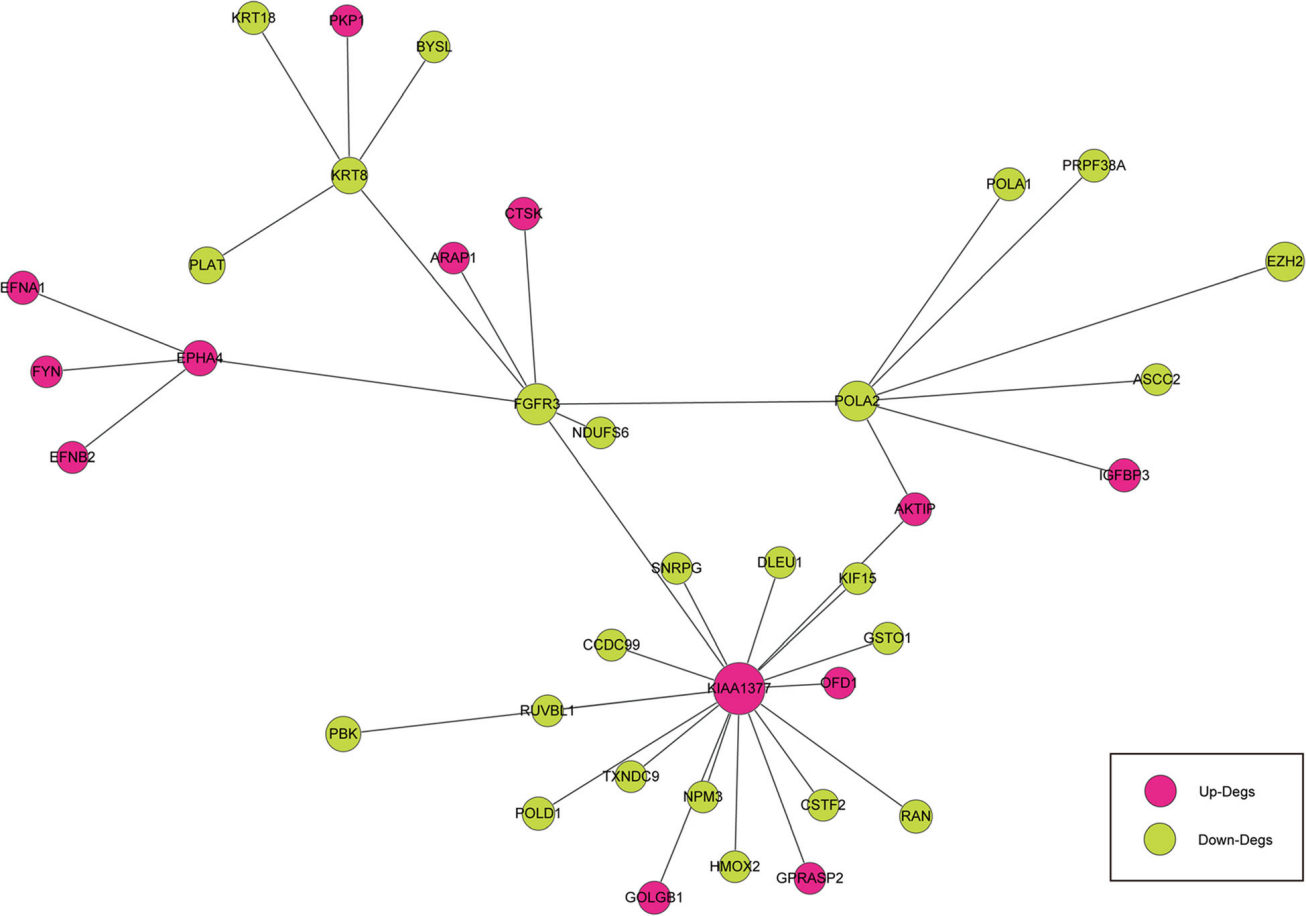
The FGFR3-centered protein–protein interaction network. The *pink* nodes represent upregulated genes, and *yellow* nodes represent downregulated genes

The genes in DEG2 group were mapped to the PPI data retrieved from the HPRD, and a network with 920 nodes and 1514 edges were created. Then, 7 DEGs, which were directly interacted with FGFR3, and other 31 interactional DEGs neighboring of these 7 nodes were selected to construct the FGFR3-centered PPI network (Fig. 1). The top 5 genes with the greatest node degree ≥4 are listed in Table 2, which were KIAA1377, POLA2 (polymerase (DNA directed), alpha 2), FGFR3, EPHA4 (ephrin type A receptor 4), and KRT8 (keratin 8).

**Table 2:** The top 5 genes with high node degree in the FGFR3-centered PPI network

Gene symbol	Average shortest	Betweenness	Degree
	Path length	Centrality	
KIAA1377	1.83333333	0.70873016	18
POLA2	2.44444444	0.27222222	7
FGFR3	1.80555556	0.61746032	7
KRT8	2.55555556	0.21269841	5
EPHA4	2.61111111	0.16190476	4

### The regulatory network related to FGFR3

**Fig. 2:**
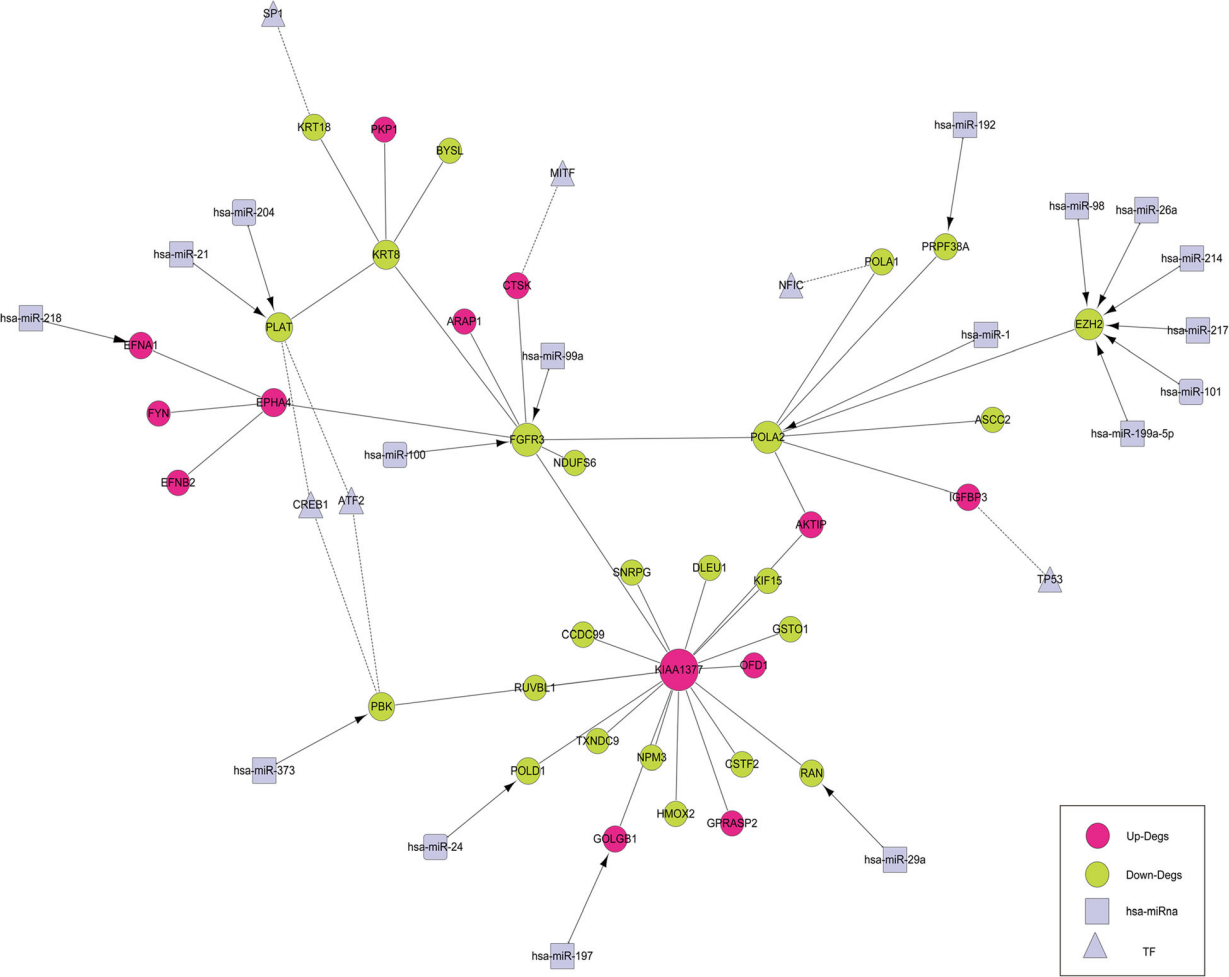
FGFR3-related regulatory network. The *pink* and *yellow* nodes represent upregulated and downregulated genes, respectively. The *square* and *triangle* nodes represent miRNAs and transcription factors, respectively. The *arrows* represent regulatory relationships between the differentially expressed genes and miRNAs while the *dotted line* represents regulatory relationships between differentially expressed genes and transcription factors

The potential regulatory miRNAs and TFs targeting to the genes included in the FGFR3-centered PPI network were screened out, and a regulatory network was constructed. There were 17 miRNAs/targets, and 8 TF/target regulatory relationships were predicted (Fig. 2).

### Prediction of potential prognostic makers for bladder cancer

**Fig. 3:**
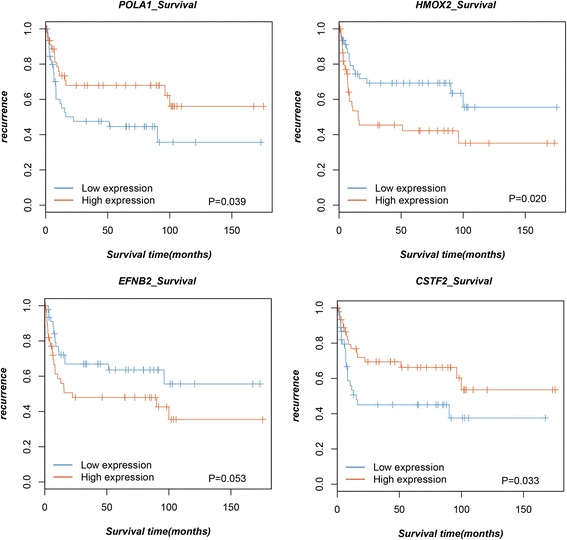
Kaplan–Meier survival curves of patients with bladder cancer according to the expression of HMOX2, CSTF2, POLA1, and EFNB2

Cox regression model was applied to identify the potential genes in the FGFR3-centered PPI network that were associated with bladder cancer prognosis. HMOX2 (heme oxygenase-2), CSTF2 (cleavage stimulation factor, 3′ pre-RNA, subunit 2, 64 kDa), and POLA1 (polymerase (DNA directed), alpha 1) were the three obviously significant related genes, and a marginal significant correlation was found between EFNB2 (ephrin-B2) and prognosis of bladder cancer (Table 3). However, a multivariate analysis about these 4 genes showed that only CSTF2 and EFNB2 were significant related to the recurrence of bladder cancer (*p* value = 0.005 and 0.030) (Table 4). Moreover, the survival curves displayed in Fig. 3 also obviously presented that patients with high expression level of CSTF2 and POLA1 showed relative favorable prognosis. However, HMOX2 and EFNB2 may be the risk factors of bladder cancer. All of these results might indicate that CSTF2 and EFNB2 were two important protective factors in the prognosis of bladder cancer.

**Table 3:** The genes significantly correlated with the prognosis of bladder cancer patients

Gene symbol	Gene ID	*p* value
HMOX2	3163	0.019917
CSTF2	1478	0.033471
POLA1	5422	0.038543
EFNB2	1948	0.053444

**Table 4:** Multivariate analysis result of 4 genes related to the prognosis of bladder cancer

Gene	*p* value	OR	95% CI
HMOX2	0.808	0.895	0.366–2.19
CSTF2	0.005	0.195	0.062–0.614
POLA1	0.536	0.685	0.206–2.271
EFNB2	0.030	2.975	1.111–7.968

## Discussion

Bladder cancer is a complex process, implicated with multiple genetic abnormalities. A body of studies evidenced that FGFR3 acts as an important oncogenic driver in bladder cancer. Here, we explored the possible mechanism how FGFR3 signaling mediates bladder cancer development and progression by bioinformatics methods. We found that the expression levels of 2855 genes were altered upon the depletion of FGFR3 in bladder cancer cell line. These downregulated genes were mainly involved in cell division and carboxylic acid metabolic process while the upregulated were related to blood vessel morphogenesis and response to type I interferon. Increased cell division is a well-known cause and essential for development and progression of human cancer [17]. Adequate blood supply, such as angiogenesis, is also required for the growth and metastasis of tumor [18]. Fibroblast growth factors (FGFs) and FGF receptors can act as angiogenesis inducers and have contribution to tumor vascularization [19]. Moreover, it has shown that IFN-a, as one type of I interferon inhibits the expression of basic fibroblast growth factor (bFGF), reduces angiogenesis and thus inhibits tumor growth in the bladder wall [20]. Therefore, FGFR3 may promote the development of bladder cancer by regulating cell division, response to type I interferon signaling, and blood vessel morphogenesis.

To gain insight into the oncogenic mechanism of FGFR3 in bladder cancer, we constructed FGFR3-related molecular networks. First, a FGFR3-centered PPI network was generated and KIAA1377, POLA2, FGFR3, EPHA4, and KRT8 were the hub genes. KIAA1377, also known as Cep126, regulates microtubule organization at the centrosome by modulating the transport of pericentriolar satellites [21]. Centrosome takes vital roles in several fundamental cellular functions, including cell division. POLA2, encoding the B-subunit of DNA polymerase α, has been reported to be involved in cell proliferation by mediating DNA replication, recombination, and repair [22]. EPHA4, a member of the EPH receptors, is closely related to tumor progression [23]. But based on the previous studies, the investigations of relationships among these hub genes were still absent. The researches of these genes were only concentrated on bioinformatics, except that EPHA4 has been confirmed to interact with FGFR3 in vitro [24]. Thus, it was very essential to explore the correlations among FGFR3, KIAA1377, POLA2, and EPHA4 in bladder cancer, as well as other cancers or diseases. Next, we predicted several regulators and constructed a regulatory network based on the PPI network. Total 17 miRNAs and 6 TFs were involved. Among these TFs, TP53 is a well-known tumor suppressor gene while MITF (microphthalmia-associated transcription factor) is an oncogene [25]. ATF2 (activating transcription factor 2) and CREB1 (CAMP responsive element binding protein 1) are both CREB-related proteins and share the same cAMP responsive element sequence. They are also involved in cancer progression [26]. From the regulatory, we found that 2 miRNAs, hsa-miR-100 and hsa-miR-99a, directly interacted with FGFR3. Song et al. have found the aberrant expression of these 2 miRNAs in bladder cancer by microarray analysis of 25 cases of bladder urothelial carcinomas and adjacent normal bladder tissue [27]. Moreover, in fact, the negative regulation of FGFR3 by mir-100 has been demonstrated in clear cell ovarian cancer cells [28]. Thus, the above molecules may be responsible for the carcinogenesis of FGFR3 in bladder cancer.

In addition to the molecule mechanism how FGFR3 contributes to the bladder cancer progression, we tried to explore the clinical value of FGFR3. Thus, we screened the bladder cancer recurrence-related genes from the FGFR3-centered PPI network. The results indicated that patients with high expression level of CSTF2 and POLA1 showed relative favorable prognosis. However, HMOX2 and EFNB2 may be the risk factors of bladder cancer. CSTF2 encodes a nuclear protein which contains a ribonucleoprotein-type binding domain in the N-terminal region. There is a significant correlation between CSTF2 and poor prognosis for lung cancer patients [29]. POLA1, encoding the catalytic subunit of DNA polymerase, is responsible for the initiation of DNA replication. Inducible HOMX1 and constitutive HOMX2 are 2 structurally related isozymes that compose heme oxygenase. The expression level of HOMX1 is associated with cervical lymph node metastasis of tongue squamous cell carcinomas [30]. EFNB2 has been evidenced to be the prognostic maker for esophageal squamous cell carcinoma [31]. In current study, we revealed that there were correlations between these 4 genes and the prognosis for patients with bladder cancer. The results further confirmed that the genes in the PPI network may be responsible for the carcinogenesis of FGFR3 in bladder cancer.

## Conclusions

In conclusion, we analyzed gene expression profiles of the bladder cancer cell line RT112 with or without depletion of FGFR3 and conducted further analyses using a computational bioinformatics approach based on the publicly available data. This study provided some insights on the mechanism underlying the carcinogenesis of FGFR3 in bladder cancer. However, further experimental studies are needed.

## Abbreviations

bFGF: Basic fibroblast growth factor
DEGs: Differentially expressed genes
FGFR3: Fibroblast growth factor receptor 3
FGFs: Fibroblast growth factors
GO: Gene Ontology
HPRD: Human Protein Reference Database
PPI: Protein–protein interaction
RMA: Robust multi-array average
RTK: Family of tyrosine kinase
